# SNP profiling of *Elaeis oleifera* (H.B.K) Cortes germplasm in Ucayali, Peru using Genotyping-by-Sequencing (GBS)

**DOI:** 10.1186/s13104-025-07336-7

**Published:** 2025-07-17

**Authors:** Alina Camacho-Villalobos, Orson Mestanza, Rosa M. Cabrera-Pintado, Glendy Sanchez Sunción, Jorge Bendezu

**Affiliations:** 1 Dirección de Desarrollo Tecnológico Agrario, Estación Experimental Agraria Pucallpa, Instituto Nacional de Innovación Agraria (INIA), Av. Centenario Km. 4 y 4.2, Coronel Portillo, 25001 Ucayali, Perú; 2 Laboratorio de Referencia Nacional de Biotecnología y Biología Molecular, Centro Nacional de Salud Pública, Instituto Nacional de Salud, Lima, Perú; 3 Dirección de Recursos Genéticos y Biotecnología, Instituto Nacional de Innovación Agraria (INIA), Lima, Perú; 4 Facultad de Ciencias Agropecuarias, Universidad Nacional de Ucayali (UNU), Coronel Portillo, Ucayali, Perú; 5 Universidad Privada del Norte, Lima, Perú

**Keywords:** *Elaeis oleifera*, American oil palm, SNP, Germplasm, Cluster, Genetic diversity

## Abstract

**Objectives:**

*Elaeis oleifera*, commonly known as the American oil palm, plays a crucial role in the agricultural economies of Central and South America due to its unique oil characteristics and resistance to certain diseases. Despite its importance, limited available genetic information has hindered the effective utilization of this species in breeding programs aimed at improving oil yield and disease resistance. This study employed Genotyping-by-Sequencing (GBS) to profile polymorphisms within *Elaeis oleifera* populations, the unique germplasm bank for Peru, located in Ucayali, Peru, aiming to characterize the gene pool.

**Data description:**

The GBS analysis successfully identified 22,703 informative single-nucleotide polymorphisms (SNPs) across the twelve-year-old plant genomes (*n* = 42). Observed heterozygosity (Ho = 0.3086) and expected heterozygosity index (He = 0.3385) were quantified. The fixation index (Fst = 0.0048) indicated low genetic differentiation within the germplasm. However, the presence of two genetic clusters (C_1 and C_2) distributed homogenously within the studied population has been detected; the origin of these clusters could be mainly associated with the initial management of the germplasm nucleus within Peru. The extensive SNP dataset provides a comprehensive genetic map that is invaluable for the conservation and enhancement of *Elaeis oleifera* in our region.

## Objective

*Elaeis oleifera*, commonly known as the American oil palm, is an essential species in the Arecaceae family, distinguished by its significant role in oil production. In Central and South America, this species contributes considerably to the agricultural economies [1–5]. For that reason, the genetic enhancement of *E. oleifera* is pivotal for improving oil yield, disease resistance, and adaptability to various environmental conditions. Genetic diversity is fundamental to crop improvement, offering the genetic variation necessary for breeding programs [6–8]. In the context of South America, the conservation and utilization of genetic diversity in crops like *E. oleifera* is crucial where the biodiversity is under constant threat from agricultural expansion and environmental changes [8, 9].

*E. oleifera* possesses several advantageous traits compared to its African counterpart, *E. guineensis*, including higher resistance to certain diseases and a more unsaturated oil profile [1, 2, 5, 9]. These traits make it an attractive candidate for breeding programs aimed at enhancing the genetic base of commercial oil palm plantations. However, the genetic improvement of *E. oleifera* is hindered by limited information on its genetic diversity and structure, especially in the South American region. Recent advancements in genomic tools have revolutionized our ability to assess and utilize genetic diversity in crop species. Genotyping-by-sequencing (GBS) is one such tool that has gained prominence for its efficiency and cost-effectiveness in generating large-scale SNP data for this crop [4, 5, 10], allowing us to explore the genome and providing a comprehensive overview of genetic variation.

A recent study on the genetic diversity and structure of American oil palm populations in the Peruvian Amazon basin employing microsatellites emphasized the importance of these genetic resources in breeding and conservation programs [2]. The study reported a high genetic variability within *E. oleifera* germplasm, underscoring the need for comprehensive genetic profiling to harness this diversity effectively.

The germplasm collection of *E.oleifera* located in Ucayali, Peru, represents a critical resource for genetic studies and breeding programs. This collection, encompassing diverse accessions from the Peruvian Amazon region (Loreto, Peru), was collected in 2010 and implemented in 2012, providing an invaluable genetic reservoir for improving oil palm traits. However, to fully exploit this potential, detailed genetic profiling is essential. This study aims to utilize GBS to profile polymorphisms within the *E.oleifera* germplasm collection. By identifying SNPs and assessing genetic diversity, we aim to elucidate the genetic structure of this collection and explore its potential to enhance breeding programs. Applying GBS in this context will not only improve our understanding of *E.oleifera* genetic diversity but also contribute to developing more resilient and productive oil palm cultivars.

## Data description

An American palm plantation, *Elaeis oleifera* H.B.K. Cortes, consisting of 42 twelve-year-old plants, was employed. The biological material is maintained at the Pacacocha annex of the Estación Experimental Agraria Pucallpa, Yarinacoha district, Coronel Portillo Provence, Ucayali region, Peru. Its geographical coordinates are 8°21 × 9.75” S and 74°33 × 5.59” W [2]. The plant material belongs to the Elaeis oleifera germplasm maintained by Estación Experimental Agraria Pucallpa, Instituto Nacional de Innovación Agraria (INIA), Peru. All materials were collected in accordance with Peruvian regulations [2].

The leaf samples were collected using BioArk leaf kit (Biosearch Technologies, Berlin, Germany) following the manufacturer’s instructions. The total DNA was extracted and quantified using the method of Doyle and Doyle with modifications [2]. The quality DNA was verified with a 1% agarose gel using a GeneRuler 1 kb DNA molecular marker (Thermo Fisher Scientific, Carlsbad, CA, USA), and were then quantified using UV-spectrophotometry in an Epoch 2 microplate spectrophotometer (Biotek, Winooski, VT, USA).

The Genotyping-by-Sequencing (GBS) protocol was followed according to a previous report [4], considering a variation in the enzyme for the library construction. In this case, the libraries were built using enzyme MsII (CAYNN/NNRTG), and paired-end read (150 bp) sequencing was performed on an Illumina™ NextSeq 500/550v2 platform at the LGC Genomics GmbH Laboratories (Berlin, Germany). The raw reads were trimmed with Trimmomatic v0.39 [11] eliminating sequences with lengths less than 30 bp. The trimmed reads were analyzed according to Stacks2 pipeline v.2.60 [12] using the reference genome E08 (GCA_000441515.1). The filter for calling SNPs was performed on VCF file with methodology reported in [4]. The obtained VCF files with high-quality filtered SNPs were used to calculate genetic diversity indices with dartRverse v1.0.2 and a tree based on UPGMA was built, to assess the genetic distance between samples with Nei’s genetic distance with 1000 bootstraps was POPPR v2.9.3 [13], were implemented in software R v4.4.1.

Our data showed in dataset 1, for the first time, the technical outcomes of GBS applied to SNP discovery and its use in genetic diversity in a Peruvian germplasm of *E. oleifera*. The paired-end sequencing of 42 samples generated a total of 18 GB of raw data. After removing low-quality sequencing reads, a mean of 1.5 million sequencing reads per sample remained. The average sequencing depth was 10×, and on average, 95.5% of sequencing reads mapped to the E08 reference genome (BioSample: SAMN02981531).

The GBS analysis successfully identified 22,703 informative SNPs. This diversity was evidenced by the broad range of genetic variations within and between the accessions studied. A high density of SNPs was observed across all chromosomes, indicating a rich genetic reservoir, with some areas showing particularly high polymorphism, possibly linked to important traits (e.g.; oil production and disease resistance). Genetic diversity indexes such as observed heterozygosity (Ho) and expected heterozygosity (He) were quantified, with values of 0.3086 and 0.3385, respectively, reflecting more genetic variability within the Peruvian germplasm than previously reported in populations from Brazil [5]. The fixation index calculated (Fst = 0.0048) suggests a low degree of genetic differentiation among samples, likely influenced by their geographic origins. Additionally, tree distance analysis (See Data file1) [14] provided insights into the genetic relationships within the Peruvian collection, aligning closely with two clusters. Finally, the genetic diversity indexes were estimated considering both groupings by clusters and geographical sites (See Data file 2 and 3) [15, 16].

These findings explained the probable existence of two genetic pools. These findings are particularly significant given the strategic locations of Jenaro Herrera and Caballococha where the plants were collected in 2010. Furthermore, Jenaro Herrera is located approximately 385.5 km northwest of Pacococha, Ucayali (germplasm location), and the other hand, Caballococha, is near the tri-border area with Colombia and Brazil at 646.0 km northwest of the germplasm location (See Data file 4) [17], showcases genetic uniqueness potentially influenced by its geographic isolation, underscoring the importance of targeted conservation strategies to preserve these unique genetic traits. Our results may be useful for genetic associations between SNPs and agricultural traits (see Table 1).Data Set 1 contains the Genotyping by sequencing of *E. oleifera* results for this publication, which have been deposited in the National Center for Biotechnology Information (NCBI) under project number PRJNA1217359 [18].

**Table 1:** Overview of data files/data sets

Label	Name of data file/data set	File types (file extension)	Data repository and identifier (DOI or accession number)
*Data file 1*	*Genetic distance tree that represents the genetic relatedness of the plants within the Peruvian Germplasm.*	*Figure 1(word file)*	*Figshare;* 10.6084/m9.figshare.28004432*(14)*
*Data file 2*	*Q-profile between population. Provide a summary of diversity measured*,* including allelic richness*,* Shannon information*,* and heterozygosity.*	*Figure 2(word file)*	*Figshare;* 10.6084/m9.figshare.28004453*(15)*
*Data file 3*	*Genetic diversity indexes (Ho*,* He*,* Ht*,* AR and Fst) for each grouping employing the Peruvian Eleais Oleifera germplasm in the Ucayali region*	*Table 2(word file)*	*Figshare;* 10.6084/m9.figshare.28004474*(16)*
*Data file 4*	*The geographical origins of the samples employed in this study are shown in the Peruvian Amazon*	*Figure 3(word file)*	*Figshare;* 10.6084/m9.figshare.26914144*(17)*
*Data set 1*	*Genotyping by sequencing of E. oleifera*	*FASTQ files*	*The National Center for Biotechnology Information (NCBI);* https://www.ncbi.nlm.nih.gov/bioproject/1217359 (18)

## Limitations

This study has some limitations. First, the analysis was based on only one germplasm collection from Ucayali, Peru, which may not represent fully represent the extensive genetic diversity present in *Elaeis oleifera* across its natural range in South America. More collections from other geographical locations would probably give a better insight into the genetic variation within the species. Second, although GBS has identified a large number of SNPs, the coverage is comparatively low, which limits the possibility of detecting rare alleles and fully characterizing complex genomic regions.

## Data Availability

The sequencing data and associated metadata from this study have been deposited in the National Center for Biotechnology Information (NCBI) under project number PRJNA1217359 (18). Please see Table 1 for details and links to the data.

## Abbreviations

SNP: Single-nucleotide polymorphism
GBS: Genotyping-by-Sequencing
